# Protrudin-mediated ER-endosome contact sites promote phagocytosis

**DOI:** 10.1007/s00018-023-04862-0

**Published:** 2023-07-19

**Authors:** Liv Anker Elfmark, Eva Maria Wenzel, Ling Wang, Nina Marie Pedersen, Harald Stenmark, Camilla Raiborg

**Affiliations:** 1 Centre for Cancer Cell Reprogramming, Faculty of Medicine, University of Oslo, Oslo, Norway; 2 Department of Molecular Cell Biology, Institute for Cancer Research, Oslo University Hospital, Oslo, Norway

**Keywords:** Protrudin, FYCO1, VAMP7, RAB7, Phagocytosis, Efferocytosis, Endosomes

## Abstract

During phagocytosis, endosomes both contribute with membrane to forming phagosomes and promote phagosome maturation. However, how these vesicles are delivered to the phagocytic cup and the phagosome has been unknown. Here, we show that Protrudin-mediated endoplasmic reticulum (ER)-endosome contact sites facilitate anterograde translocation of FYCO1 and VAMP7-positive late endosomes and lysosomes (LELys) to forming phagocytic cups in a retinal pigment epithelial-derived cell line (RPE1). Protrudin-dependent phagocytic cup formation required SYT7, which promotes fusion of LELys with the plasma membrane. RPE1 cells perform phagocytosis of dead cells (efferocytosis) that expose phosphatidylserine (PS) on their surface. Exogenous addition of apoptotic bodies increased the formation of phagocytic cups, which further increased when Protrudin was overexpressed. Overexpression of Protrudin also led to elevated uptake of silica beads coated with PS. Conversely, Protrudin depletion or abrogation of ER-endosome contact sites inhibited phagocytic cup formation resulting in reduced uptake of PS-coated beads. Thus, the Protrudin pathway delivers endosomes to facilitate formation of the phagocytic cup important for PS-dependent phagocytosis.

**Supplementary Information:**

The online version contains supplementary material available at 10.1007/s00018-023-04862-0.

## Introduction

Phagocytosis is defined as a receptor-mediated engulfment of large particles (≥ 0.5 µm) such as pathogens and dead cells. Upon binding to a particle, the plasma membrane (PM) will form pseudopods, also called a phagocytic cup, to surround and engulf the object [1]. Non-professional phagocytes such as epithelial cells, endothelial cells and fibroblasts can remove dead cells, in a process termed efferocytosis [2, 3]. Dying or dead cells expose various forms of the lipid phosphatidylserine (PS), which is recognised by receptors on the phagocyte. In this manner, phagocytes can discriminate between living and dead cells [4, 5]. Defects in the efferocytosis machinery can lead to autoimmune diseases, cancer, and neurodegenerative diseases [6–10]. Some organs rely on efferocytosis for proper function, like the eye’s retina, where retinal pigment epithelial (RPE) cells daily engulf photoreceptor outer segments to avoid blindness [11, 12].

To cope with engulfment of relatively large particles such as apoptotic bodies, the cell can instruct intracellular membranes to fuse with the PM to increase the surface area by focal exocytosis [13–15]. Recycling endosomes containing the SNARE protein VAMP3 [16] and late endosomes and lysosomes (LELys) positive for TI-VAMP/VAMP7 (VAMP7) [17] are known membrane sources for phagocytic cup formation. During phagocytosis, VAMP7-mediated fusion of endosomes with the PM is dependent on Ca^2+^ release from lysosomes [18] and regulated by the Ca^2+^ sensor Synaptotagmin VII (SYT7), which is enriched on lysosomes [19, 20]. Despite substantial knowledge about the internal membrane sources in phagocytosis [21], the mechanism behind the translocation of vesicles to forming phagosomes is not known.

One intracellular membrane transportation pathway known to translocate endosomes from the cell centre to the periphery is the Protrudin pathway. The endoplasmic reticulum (ER)-residing protein, Protrudin, and its associated protein partners promote anterograde transport of LELys to the PM in neurite outgrowth, axonal regeneration and membrane protrusions formed by cancer cells, so-called invadopodia [22–25]. Protrudin forms ER-endosome contact sites by binding to the small GTPase RAB7 and phosphatidylinositol 3-phosphate (PtdIns3P) in the LELys membrane. Upon contact site formation, Protrudin supplies the LELys with the microtubule motor protein Kinesin-1, which binds to the endosomal Kinesin-1 adapter protein, FYVE and Coiled-Coil Domain Autophagy Adaptor 1 (FYCO1). By repeating this step frequently during transportation, LELys can travel along microtubules in a plus-end direction where they eventually can fuse with the PM [24]. Analogous to phagocytosis, the fusion is dependent on SYT7 to support neurite outgrowth in PC12 cells and invadopodium formation in MDA-MB-231 cells [24, 25]. Interestingly, VAMP7-positive LELys are also recruited to invadopodia [26], but this has not been studied in the context of Protrudin-dependent vesicle transport.

Both invadopodia and phagocytic cups are actin-rich structures protruding from the cellular surface that rely on endosome translocation and focal exocytosis. Due to their molecular similarities and Protrudin’s role in PM remodelling through anterograde endosome transport in various cell types, we hypothesised that this pathway could be involved in vesicle transport in phagocytosis. Here, we show that the Protrudin pathway is required for efferocytosis in the immortalised RPE cell line (RPE1), where it stimulates phagocytic cup formation and the uptake of phagocytic cargo.

## Results

### Protrudin overexpression increases phagocytic cup formation

**Fig. 1:**
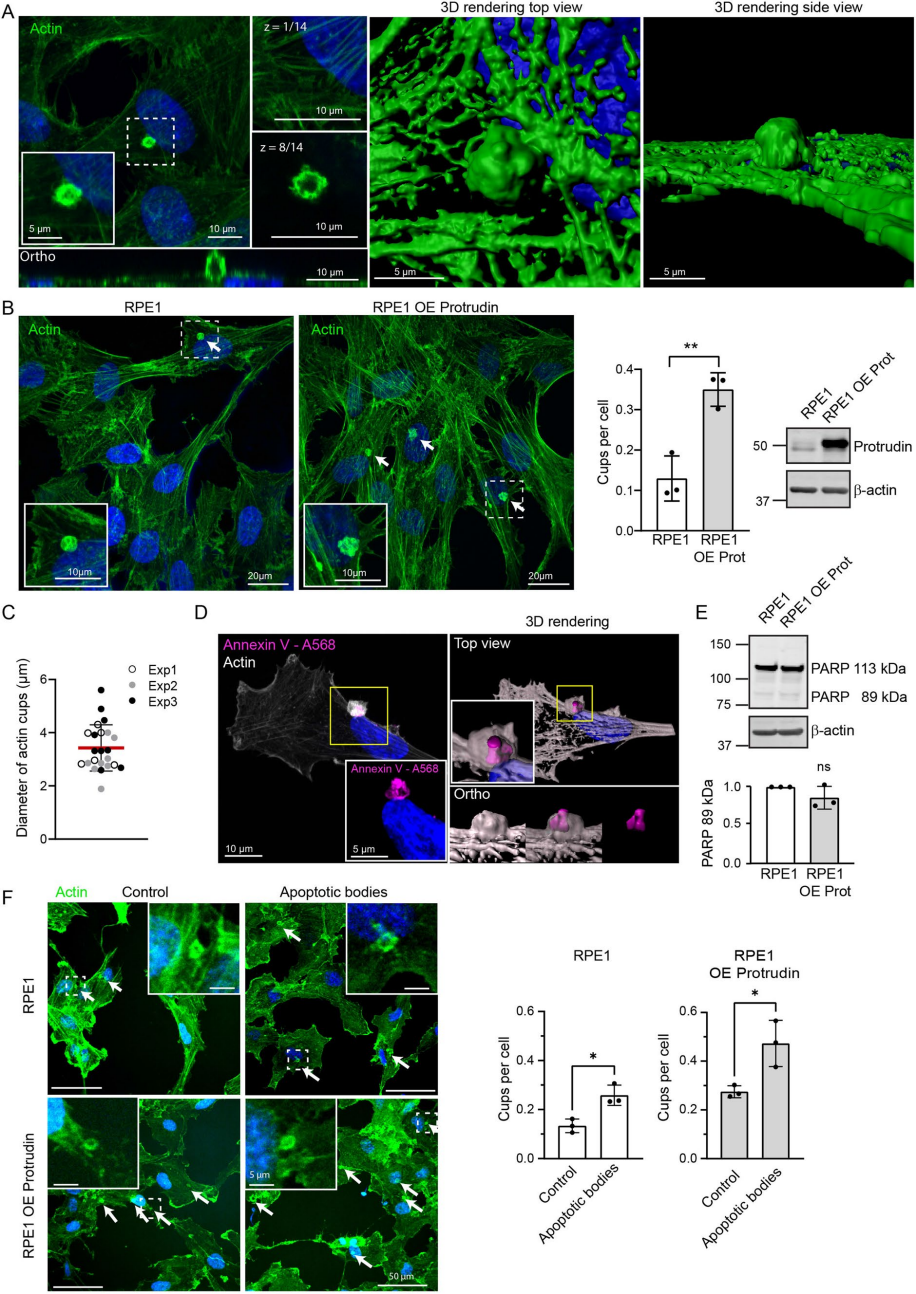
Overexpression of Protrudin promotes phagocytic cup formation. **A** RPE1 cells were grown on coverslips, fixed, and stained with Phalloidin Alexa Fluor 488 to visualise F-actin and imaged by confocal microscopy. Shown is the sum projection of a confocal z-stack. Right insets display the actin organisation in two confocal sections, where the highest section shows an actin-rich circular structure. “z = ” indicates which optical confocal section from the z-stack is displayed. The bottom panel shows the orthogonal view of the actin structure. Images to the right are 3D renderings of the same structure shown from two different angles. **B** RPE1 and RPE1 OE Protrudin cells were grown on coverslips, fixed, and stained with Phalloidin Alexa Fluor 488. A confocal z-stack is displayed as a maximum intensity projection. Arrows point to actin cups. The number of actin cups is increased when Protrudin is overexpressed. Bar graph shows the mean number of actin cups per cell ± s.d, analysing over 200 cells in each experiment, *n* = 3 experiments. **P < 0.01, unpaired two-tailed *t* test. Western blot validating the expression level of Protrudin in the RPE1 cells and the stable cell line overexpressing Protrudin. β-actin acts as a loading control. Note that RPE1 cells express several Protrudin isoforms often represented as a double band around 50 kDa. Exogenous Protrudin in RPE1 OE cells is the canonical isoform 1. *OE Prot* overexpressed Protrudin. **C** Quantification of the diameter of dorsal actin structures from RPE1 cells. Each dot represents one measurement colour coded by experimental replicate. 23 cups were analysed in total. Graph denotes mean ± s.d., *n* = 3 experiments. **D** Confocal images of RPE1 cells that were fixed and stained with AnnexinV-Alexa-568 and Phalloidin Alexa Fluor 488 to visualise phosphatidylserine-rich particles and F-actin, respectively. Shown is a maximum intensity projection of a confocal z-stack with a close-up showing the enrichment of AnnexinV-Alexa-568 inside the actin structure. A 3D rendering shows the same structure from different angles. Displayed is one representative image of at least 5 micrographs taken per experiment, *n* = 3 experiments. **E** Western blot of whole cell lysate from untreated RPE1 and RPE1 OE Protrudin cells, showing a strong band of full-length PARP (113 kDa). A weak band below represents a small amount of cleaved PARP (89 kDa) present in the culture. Representative lysate from three experiments, β-actin is used as a loading control. OE Prot = Overexpressed Protrudin. The graph represents the quantification of cleaved PARP (89 kDa). Shown is mean ± s.d., *n* = 3 experiments. *ns* not statistically significant, one sample *t* test. **F** RPE1 cells or RPE1 OE Protrudin cells were treated with apoptotic bodies or complete medium as a negative control and stained with Phalloidin Alexa Fluor 488. Addition of apoptotic bodies increases the number of actin cups in both cell lines. The graphs represent the mean number of cups per cell ± s.d. More than 200 cells were counted per condition, in each experiment,* n* = 3 experiments. *P < 0.05, unpaired two-tailed *t* test

When the cultured RPE1 cell line was stained with Phalloidin for fluorescence imaging, actin-rich circular structures were frequently observed on the surface of the cells. These structures were tall, hollow, rounded and always protruding on the dorsal side (Fig. 1A). Interestingly, stable overexpression (OE) of Protrudin in RPE1 cells (RPE1 OE Protrudin) led to a significant increase in the number of these structures (Fig. 1B, Supplementary Fig. 1A). The diameter of the cups was approximately 2–5 µm (Fig. 1C) corresponding to typical sizes of apoptotic bodies [27]. To analyse the content of the cups, RPE1 cells were fixed and stained with Alexa-568-conjugated Annexin V (AnnexinV-Alexa-568), a protein with high affinity for phosphatidylserine (PS), a lipid exposed on the outer membrane of apoptotic cells. Indeed, the actin-dense structures contained particles enriched in AnnexinV-Alexa-568 (Fig. 1D). Taken together, the actin-rich structures probably represent phagocytic cups engulfing apoptotic bodies present in the cell culture due to cellular turnover.

To exclude that the elevated number of phagocytic cups in the RPE1 OE Protrudin cells was due to increased apoptosis in these cells, we analysed the levels of a marker of apoptosis by Western blotting. The full-length version of the protein poly (ADP-ribose) polymerase (PARP) has the molecular weight of 113 kDa. During apoptosis, PARP is cleaved into an 89 kDa fragment and a 24 kDa fragment [28]. Western blot analysis of lysates from RPE1 and RPE1 OE Protrudin cells showed that both cell lines had strong bands for the full-length PARP, and only barely detectable bands for the 89 kDa cleaved PARP (Fig. 1E). The faint band of cleaved PARP could account for the observed efferocytosis in the cultured RPE1 cells, even though the population of dead cells was minute. Importantly, the RPE1 OE Protrudin cell line did not have a larger population of dead cells compared to the parental line. This suggests that it is indeed the higher level of Protrudin that promotes the increased amount of phagocytic cups.

As the low level of apoptosis (Fig. 1E) correlated with the low frequency of phagocytic cup formation observed in the RPE1 cells (Fig. 1B), we asked whether their formation could be stimulated by exogenous addition of apoptotic bodies. To address this question, apoptotic particles were generated by treating RPE1 cells with the apoptosis inducer Staurosporine [29]. Apoptotic bodies collected from the medium were concentrated and validated by Western blotting showing a distinct band of 89 kDa cleaved PARP (Supplementary Fig. 1B, C). Both RPE1 cells and RPE1 OE Protrudin cells showed a two-fold increase in the number of phagocytic cups after treatment with the concentrated apoptotic bodies compared to untreated cells (Fig. 1F). In summary, our data point to a positive role of the Protrudin pathway in phagocytic cup formation.

### Protrudin-induced cup formation depends on functional ER-endosome contact sites

**Fig. 2:**
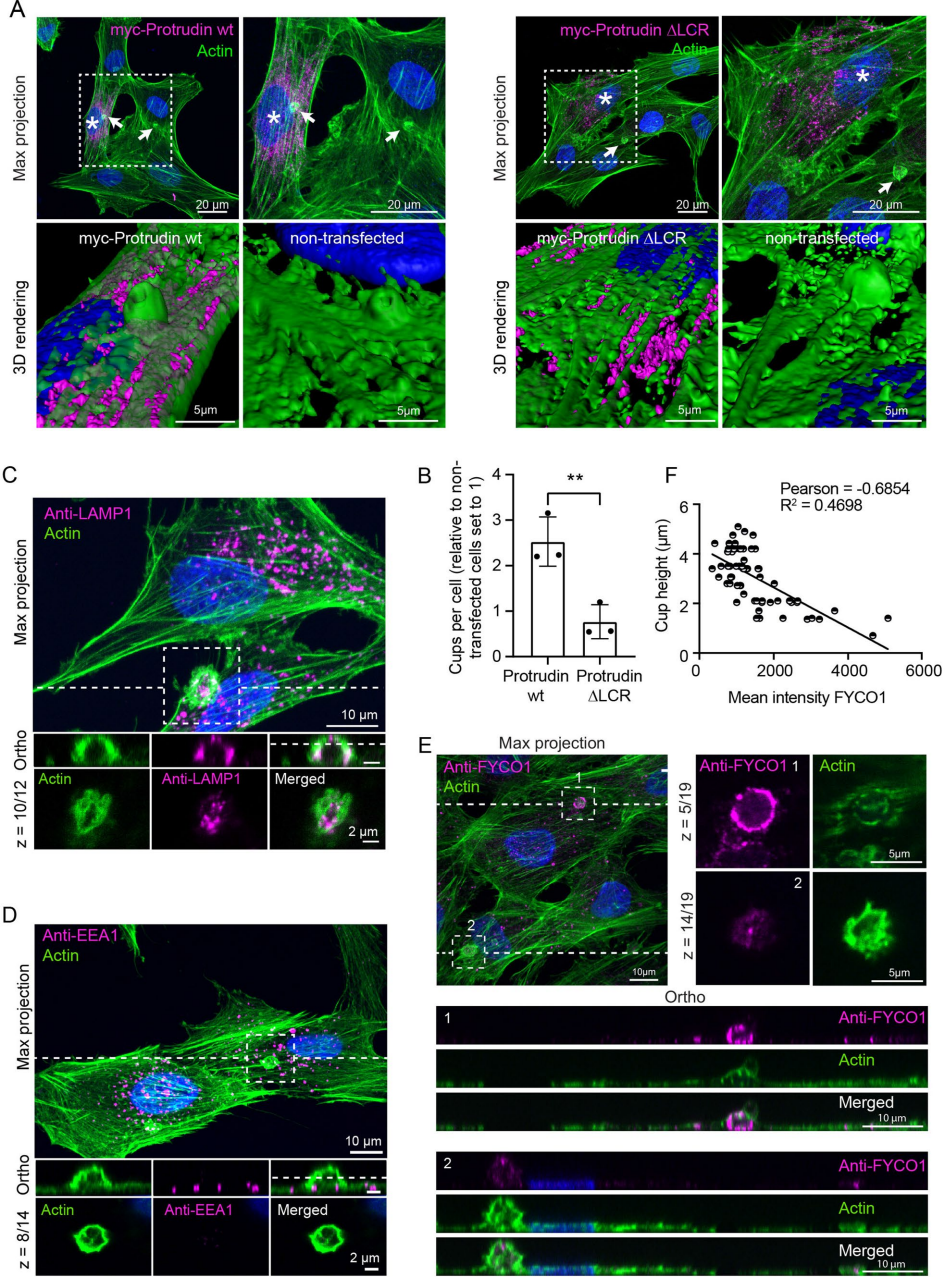
Protrudin-mediated ER-endosome contact sites are required for the formation of phagocytic cups enriched in FYCO1-positive LELys. **A** Representative confocal micrographs showing RPE1 cells transiently transfected with myc-Protrudin wt or ∆LCR, deficient in RAB7 binding. Note that the myc-Protrudin ∆LCR expressing cell does not form a phagocytic cup as opposed to myc-Protrudin wt or non-transfected cells. Phalloidin Rhodamine (green), anti-myc (magenta). Asterisks indicate transfected cells. **B** Quantification of the relative amount of phagocytic cups found in myc-Protrudin wt or ∆LCR expressing RPE1 cells, as shown in A. The graph represents mean ± s.d, *n* = 3 experiments, unpaired two-tailed *t* test. **P < 0.01. In total, 360 cells were quantified for each condition. **C**, **D** Representative confocal micrographs of RPE1 OE Protrudin cells stained with Phalloidin Alexa Fluor 488 and either anti-LAMP1 or anti-EEA1 antibodies. Maximum intensity projection and zoom-in on phagocytic cups. An orthogonal view of the highlighted area and a single confocal section are displayed. “z = ” indicates which optical confocal section from the z-stack is displayed. Note that LAMP1, but not EEA1 is enriched in the phagocytic cup. Representative image of 5 images per experiment, *n* = 3 experiments. **E** RPE1 OE Protrudin cells were seeded on coverslips, fixed, and analysed by confocal microscopy. Cells were stained with an antibody against FYCO1 and with Phalloidin Alexa Fluor 488. A maximum intensity projection of a confocal z-stack shows two phagocytic cups, and the orthogonal sections display the different cup heights. Zoom-ins highlight two confocal sections of the indicated regions. “z = ” indicates which optical confocal section from the z-stack is displayed. Note that FYCO1 fluorescence intensities differ. **F** Graph demonstrates an inverse correlation between the height of the actin cup and the mean intensity of FYCO1 staining. Intensity was measured in a ROI around each actin cup from the sum projections of confocal z-stacks. Cup height was calculated by adding all the confocal sections showing the same actin circle and multiplying with the step size of 0.34 μm. Each plotted point represents one cup, and the line represents the linear regression with a *R*^2^ = 0.4698, Pearson correlation = − 0.6854. A total of 63 cups were quantified, *n* = 2 experiments

Protrudin-mediated ER-endosome contact sites depend on the interaction between Protrudin in the ER and RAB7 in the LELys membrane, where Protrudin interacts directly with RAB7-GTP through its low complexity region (LCR) [24]. These ER-endosome contact sites are a prerequisite for Protrudin-mediated anterograde LELys translocation [24]. To test whether functional ER-endosome contact sites were required for phagocytic cup formation, we transfected RPE1 cells with myc-Protrudin wild type (wt) or a mutant where the LCR domain was deleted (∆LCR). In line with previous results [24], Protrudin ∆LCR failed to establish contact with RAB7-positive LELys, which localised in the perinuclear area as opposed to the more peripherally localising RAB7-LELys in Protrudin wt expressing cells (Supplementary Fig. 2). Importantly, Protrudin ∆LCR was not able to induce phagocytic cup formation to the same extent as Protrudin wt (Fig. 2A, B). This indicates that Protrudin-RAB7-mediated ER-endosome contact sites facilitate phagocytic cup formation and implies that Protrudin-mediated transport of RAB7-positive LELys plays an important role.

### FYCO1 and RAB7-positive vesicles are recruited to the phagocytic cup

In Protrudin-mediated ER-endosome contact sites, Kinesin-1 is transferred from Protrudin to the LELys [24]. Being fuelled with a motor protein, the LELy is then released from the ER and translocates along microtubules to the cell periphery. To characterise the population of endosomes recruited to the phagocytic cup in RPE1 cells, immunofluorescence staining for different endosomal markers was performed. Immunofluorescence micrographs showed that LELys with lysosomal associated membrane protein 1 (LAMP1) were clearly recruited to the phagocytic cup (Fig. 2C). In contrast, vesicles with the early endosomal marker early endosome antigen 1 (EEA1) could be found nearby but were not enriched in the cup (Fig. 2D). This could indicate a preferential recruitment of LELys over early endosomes to the phagocytic cup.

The Kinesin-1 adaptor FYCO1 is found on a subpopulation of LELys and is a key protein in the Protrudin pathway. Consistent with previous reports using other cell lines [24, 25, 30], we observed that FYCO1 localised to RAB7-positive LELys in RPE1 cells (Supplementary Fig. 3A). Interestingly, FYCO1-positive LELys were enriched in the phagocytic cups together with the LELys markers RAB7 and LAMP1, but not with the early endosome marker EEA1 (Supplementary Fig. 3B). In addition, we observed an even stronger recruitment of FYCO1 vesicles in actin-light cups (Fig. 2E). A quantification comparing the height of the phagocytic cup (using actin as a marker) and the total fluorescence intensity of FYCO1 demonstrated that FYCO1-positive vesicles were more enriched in the shallowest cups (Fig. 2F). Taken together, this implicates that the Protrudin pathway can deliver RAB7 and FYCO1-positive LELys to the cup, preferentially to a stage where actin is less polymerised.

### Knockdown of Protrudin or SYT7 decreases the formation of phagocytic cups

**Fig. 3:**
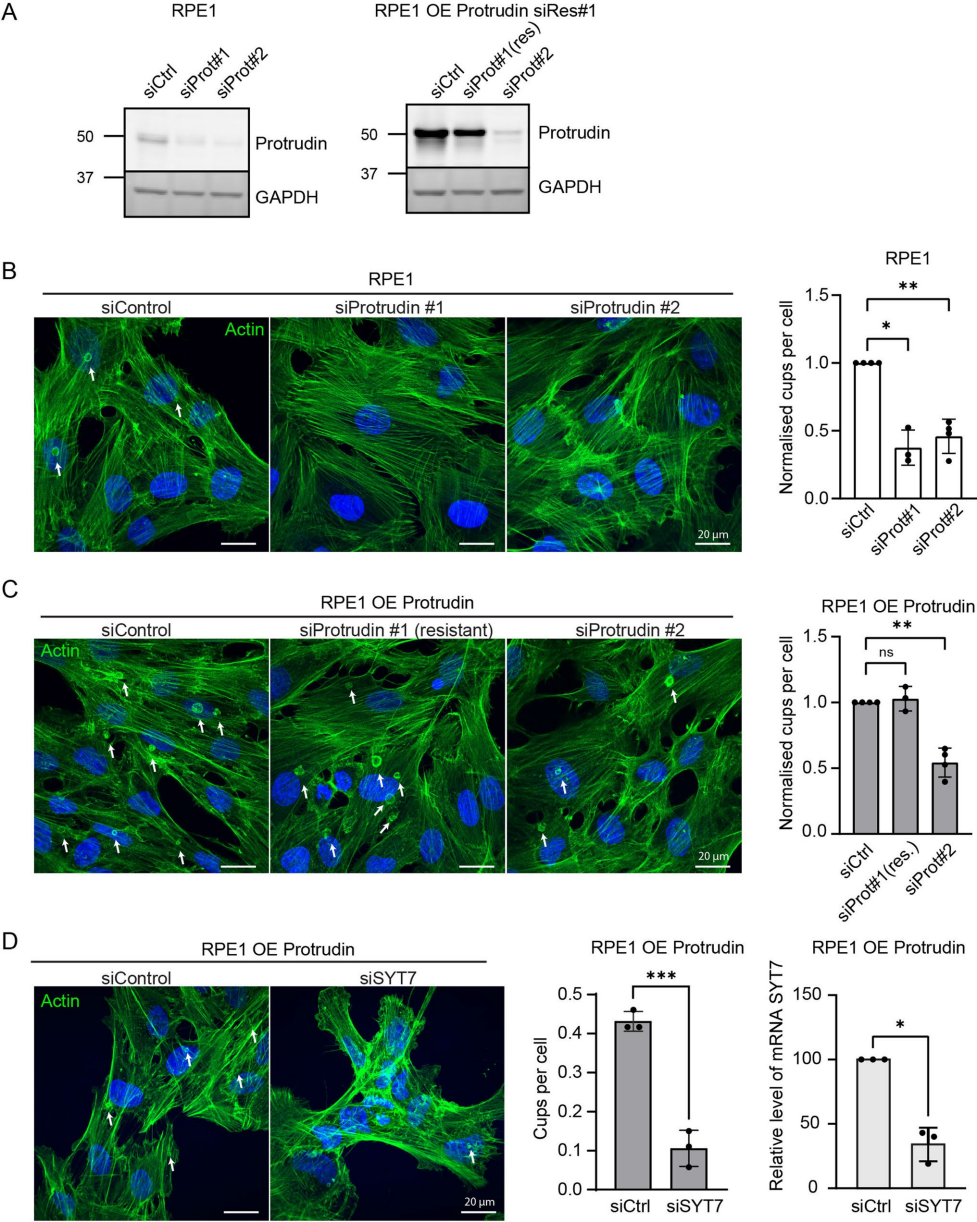
Protrudin or SYT7 depletion decreases formation of phagocytic cups. **A** Western blots showing the efficiency of siRNA depletion of Protrudin in RPE1 cells and RPE1 OE Protrudin cells. GAPDH was used as loading control. siCtrl = siRNA control, siProt#1 = siRNA Protrudin oligonucleotide 1, siProt#2 = siRNA Protrudin oligonucleotide 2, res. = resistant. **B**, **C** Representative maximum intensity projections of confocal z-stacks of RPE1 cells or RPE1 OE Protrudin cells stained with Phalloidin Rhodamine (green). Arrows point to phagocytic cups. Depletion of Protrudin with siRNA oligonucleotides hinders the formation of phagocytic cups in both RPE1 and RPE1 OE Protrudin cells. The phenotype of phagocytic cup formation in the OE Protrudin cell line was rescued in cells expressing Protrudin resistant against siRNA Protrudin #1. Graph demonstrates the mean number of phagocytic cups per cell, comparing the various siRNA treatments and cell lines, depicted as fold change of siRNA control. Each bar represents the mean ± s.d. of *n* = 3 or 4 experiments, analysing > 200 cells per experiment. *P < 0.05, **P < 0.01, n.s. not significant, one sample *t* test. **D** RPE1 OE Protrudin cells were treated with siRNA against SYT7 or siControl and analysed after 48 h. The cells were fixed and stained with Phalloidin Rhodamine to be analysed by confocal microscopy. Arrows indicate phagocytic cups in siRNA control and siRNA SYT7 treated cells in maximum intensity projections of confocal z-stacks. SYT7 depletion reduced the formation of phagocytic cups. Values denote mean ± s.d. cups per cell for more than 100 cells analysed in each experiment, *n* = 3 experiments. ***P < 0.001, unpaired two-tailed *t* test. Real-time PCR for verification of SYT7 knockdown from three independent experiments in RPE1 cells overexpressing Protrudin. *P < 0.05, one sample *t* test

As overexpression of Protrudin in RPE1 cells led to an increase in phagocytic cups, we characterised the effect of a reduced level of Protrudin (Fig. 3A, Supplementary Fig. 4A). siRNA-mediated depletion of Protrudin in RPE1 cells by two independent siRNA oligos considerably reduced the number of phagocytic cups (Fig. 3B). In line with this, Protrudin depletion in RPE1 OE Protrudin cells significantly reduced the elevated number of cups observed in these cells (Fig. 3C). Importantly, this effect was rescued in the RPE1 OE Protrudin cell line, which is resistant to siRNA oligo 1 (Fig. 3A, C, Supplementary Fig. 4A). These results point to a specific role of Protrudin in the formation of phagocytic cups in RPE1 cells.

Protrudin-mediated protrusion formation depends on LELys translocation to the cell periphery and subsequent SYT7-mediated fusion with the PM [24, 25]. Interestingly, SYT7 has similarly been implicated in focal exocytosis of endosomes during phagocytosis [19]. To test if the Protrudin-induced formation of phagocytic cups required SYT7 for fusion, we depleted SYT7 in the RPE1 OE Protrudin cells by siRNA. Notably, phagocytic cup formation was virtually abolished upon SYT7 depletion in these cells (Fig. 3D). From this experiment, we cannot exclude that other translocation pathways also deliver SYT7-loaded LELys to the phagocytic cup. However, since SYT7-mediated focal exocytosis acts downstream of Protrudin-mediated endosome translocation, our findings are consistent with an involvement of the Protrudin pathway in phagocytic cup formation.

### Protrudin promotes translocation of FYCO1 and VAMP7-positive vesicles to the cell periphery

**Fig. 4:**
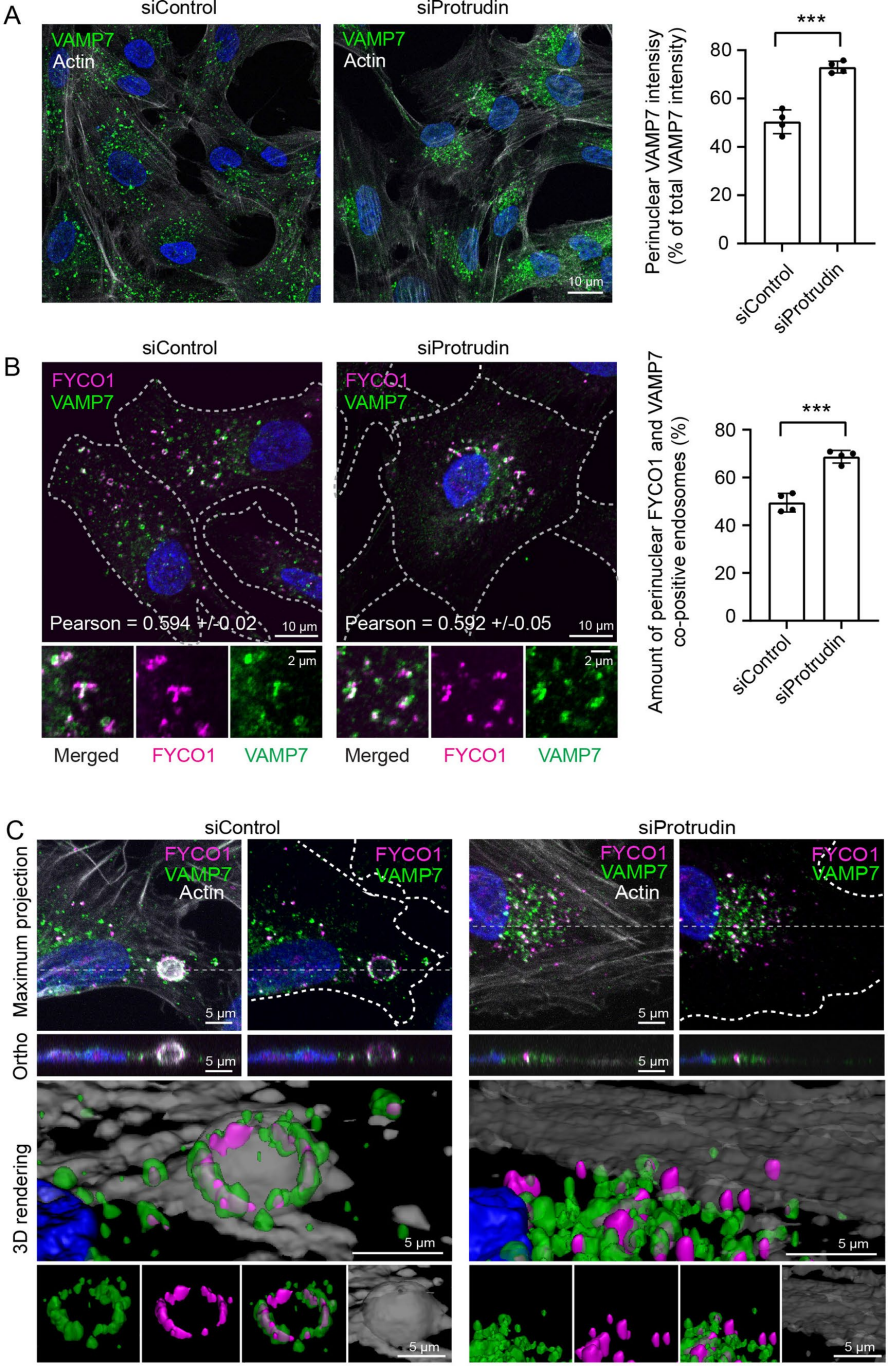
Recruitment of FYCO1 and VAMP7-positive endosomes to the phagocytic cup requires Protrudin. **A** RPE1 cells were treated with siRNA Protrudin #1 and seeded on coverslips. 48 h after transfection the cells were fixed and stained with anti-VAMP7 antibody and Phalloidin Alexa Fluor 647. In the Protrudin depleted cells, VAMP7-positive endosomes cluster more perinuclearly, implicating a role for Protrudin in VAMP7-positive endosome positioning. Representative images of a minimum of 10 images per experiment, *n* = 4. The graph represents the relative sum fluorescence intensity of VAMP7-positive puncta in the perinuclear region as % of the complete cellular population of VAMP7 dots from at least 190 cells per condition quantified using the NisElements software. Shown is mean ± s.d., *n* = 4 experiments ***P < 0.001, unpaired two-tailed *t* test. **B** RPE1 cells were treated as in A and stained with anti-VAMP7 and anti-FYCO1 antibodies. Numbers denote Pearson correlation coefficient calculated from 5 images per condition. Insets highlight the co-occurrence of VAMP7 and FYCO1 on the same endosomal structures. The graph represents the quantification of the degree of perinuclear positioning of FYCO1 and VAMP7 co-positive dots detected automatically using NisElements. Shown is mean ± s.d., n = 4 experiments ***P < 0.001, unpaired two-tailed *t* test. > 190 cells were quantified per condition, same dataset as in A. **C** Maximum projection, orthographic view and 3D rendering of confocal z-stacks showing VAMP7, FYCO1 and F-actin in RPE1 cells transfected with control siRNA or Protrudin siRNA. Note that FYCO1 and VAMP7 co-positive dots accumulate at the base of an actin-rich cup in the control treated cells, whereas the Protrudin depleted cell does not form a cup and shows a perinuclear localization of FYCO1 and VAMP7.

**Fig. 5:**
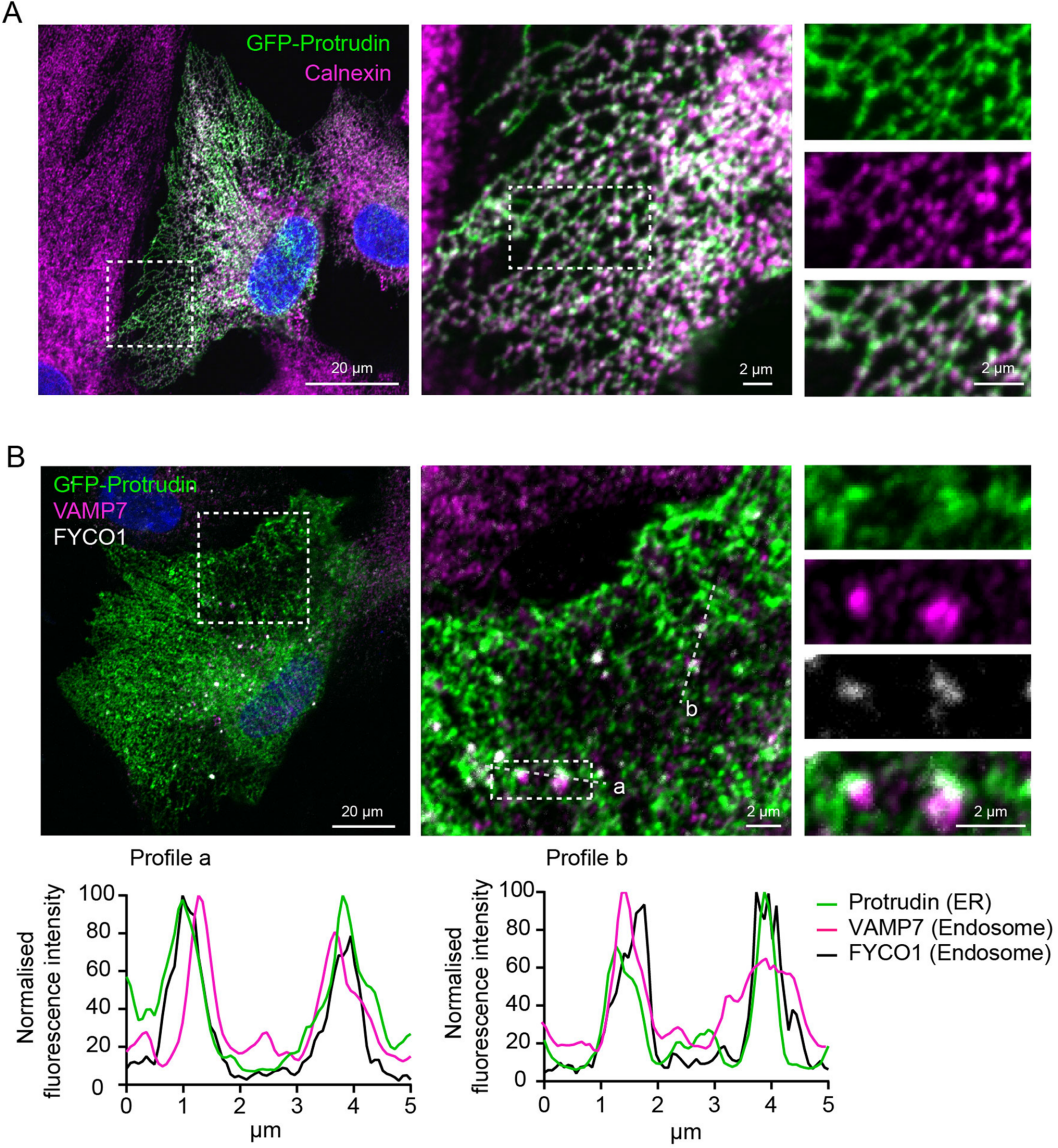
Protrudin colocalises with Calnexin in the ER and forms contact sites with VAMP7 and FYCO1-positive LELys. **A** RPE1 cells were transiently transfected with GFP-Protrudin, fixed and stained with antibodies against GFP and the ER marker Calnexin, showing that Protrudin resides in the ER. Representative of 12 micrographs. **B** RPE1 cells were transiently transfected with GFP-Protrudin, fixed and stained with anti-VAMP7 and anti-FYCO1 antibodies. The insets highlight two VAMP7 and FYCO1 co-positive dots in close apposition to GFP-Protrudin in the ER. The fluorescence intensity line plots give two examples of how VAMP7 and FYCO1-positive dots overlap with Protrudin in the ER, indicative of ER-endosome contact sites

Previous research has shown that LELys which fuse with the PM during phagocytic cup formation contain the transmembrane SNARE protein VAMP7 [17]. VAMP7 can therefore be utilised as a marker to track LELys destined specifically for focal exocytosis in the phagocytic cup. To investigate whether VAMP7 could be used as a reporter for Protrudin-mediated LELys transport, we checked if siRNA depletion of Protrudin would affect the subcellular localisation pattern of VAMP7-positive endosomes. To this end, we performed immunofluorescence imaging with an anti-VAMP7 antibody, which has been thoroughly validated elsewhere [31]. In control cells, VAMP7-positive endosomes were found perinuclearly, distributed in the cytosol and along the edges of the cell (Fig. 4A). In Protrudin depleted cells, endosomes containing VAMP7 preferably clustered around the nucleus and were not observed near the borders of the cell (Fig. 4A, Supplementary Fig. 4B). Moreover, FYCO1 localised to a substantial subpopulation of the VAMP7-positive endosomes in the presence or absence of Protrudin (Fig. 4B). In Protrudin depleted cells, the FYCO1 and VAMP7 co-positive LELys clustered in the perinuclear area (Fig. 4B, C). This implies that the Protrudin pathway is important for the transportation of FYCO1 and VAMP7-positive LELys to the periphery of the cell and that without Protrudin, these LELys have a significantly reduced capacity to reach the PM. Thus, the lack of phagocytic cups observed in Protrudin depleted cells (Fig. 3B) can be explained by impaired transport of FYCO1 and VAMP7-positive LELys to the PM (Fig. 4B, C). Indeed, FYCO1 and VAMP7-positive LELys made contact with Protrudin in the ER, in line with a role of the Protrudin pathway in the translocation of FYCO1 and VAMP7-positive LELys to the phagocytic cup (Fig. 5A, B).

### FYCO1 and VAMP7-positive vesicles are recruited during phagocytic cup completion

**Fig. 6:**
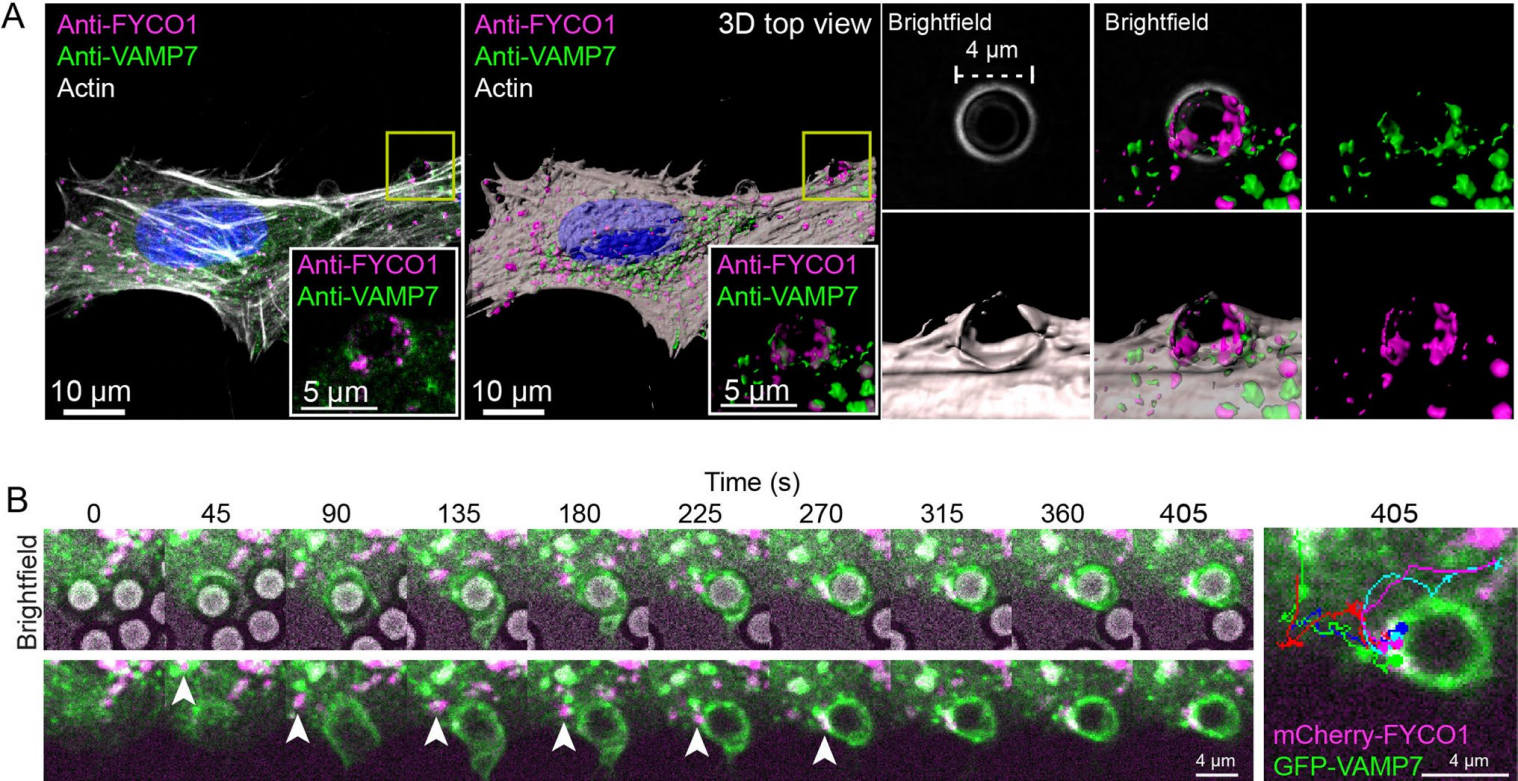
VAMP7 and FYCO1-positive LELys are recruited to phagocytic cups as they form. **A** RPE1 cells were incubated with 4 μm diameter PS-beads for 15 min at 37 °C before fixation. Cells were immunostained with anti-VAMP7, anti-FYCO1 and Phalloidin Rhodamine. Micrograph of a cell (maximum intensity projection) with double-positive endosomes enriched around a PS-bead. 3D surface rendering of the same image shows the cell from the top. Insets show the endosomes and/or actin in the highlighted area with or without the bead in brightfield. Representative image from a total of 16 confocal z-stacks, *n* = 4 experiments. **B** RPE1 cells were transiently transfected with GFP-VAMP7 and mCherry-FYCO1 for 24 h before live-cell imaging. PS-beads were added to the cells and the montage (still images from Video 1) shows close-up frames from every 45^th^ second in a cell where bead uptake occurs. The bead is visible in brightfield. Arrowhead highlights one FYCO1 and VAMP7 double-positive endosome transported to the bead during engulfment. The image to the right visualises tracks from endosomes traveling towards the phagocytic cup. Each coloured line represents the transportation route of one double-positive endosome to the phagocytic cup, arrowheads in the montage correspond to the green track

To generate an expedient experimental design that allowed us to study endosome recruitment in the phagocytic cup, cells were incubated with silica beads that were coated with PS-containing liposomes (PS-beads) for 15 min before fixation and immunostaining. Confocal immunofluorescence microscopy revealed endosomes positive for both VAMP7 and FYCO1 in close apposition to the PS-beads in actin-rich cups at the cell surface (Fig. 6A). In these regions, VAMP7 was present both together with FYCO1 and on separate endosomes.

To investigate the dynamics of LELys recruitment during the completion of the phagocytic cup, we performed live cell imaging of RPE1 cells transiently expressing mCherry-FYCO1 and GFP-VAMP7. FYCO1 and VAMP7-positive LELys were recruited to the base of the phagocytic cup during PS-bead internalization (Fig. 6B, Video 1). The vesicles appeared to fuse with the PM as indicated by an increase in the GFP-VAMP7 signal in the forming phagocytic cup, which eventually surrounded the PS-bead. Taken together, our data support a role of Protrudin-mediated endosome delivery during the completion of the phagocytic cup.

### Protrudin promotes phagocytosis of PS-rich particles

To investigate whether Protrudin is required for phagocytic uptake of the PS-beads, we first tested whether the beads could be fully internalised into the cells. Live cell imaging of RPE1 cells transiently transfected with pHluorin-LAMP1-mCherry showed that the internalised PS-beads became positive for pHluorin-LAMP1-mCherry (Supplementary Fig. 5A, Video 2). Importantly, the pH sensitive pHluorin signal was lost as the phagocytosed beads moved towards the cell centre, indicative of phagosome acidification and maturation. Thus, the PS-beads can be used to study the role of Protrudin in phagocytosis.

**Fig. 7:**
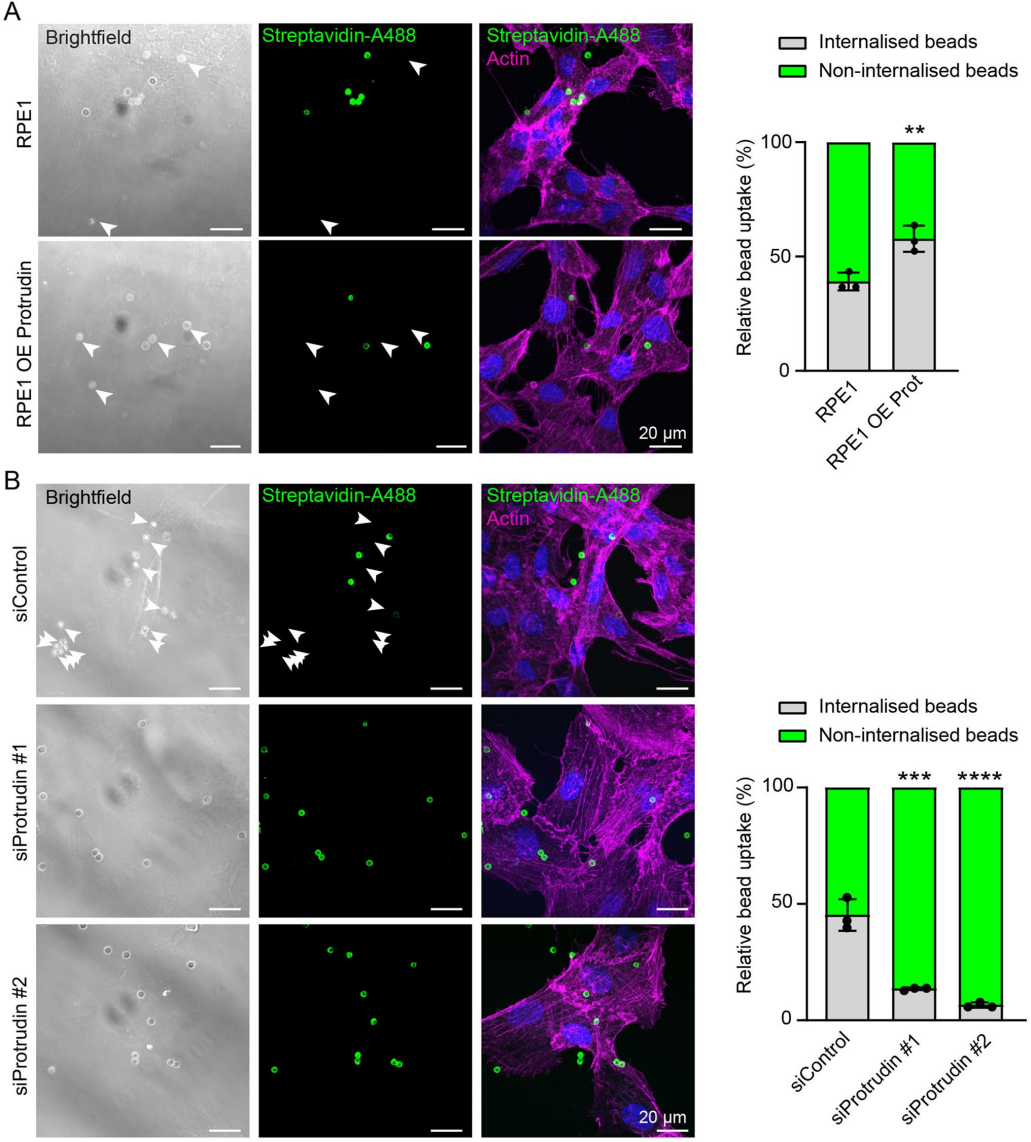
Protrudin expression levels affect the uptake of PS-beads. **A** RPE1 and RPE1 OE Protrudin cells seeded on coverslips were incubated with PS-biotin beads for 30 min. Streptavidin-Alexa-488 labelling allows to distinguish non-internalised from internalised beads (see methods section). Maximum intensity projections of representative z-stacks show that the OE Protrudin cells have more internalised beads (arrows) compared to parental cells. Graph shows the relative percentage of internalised beads to the number of total cell-associated beads. Values denote mean ± s.d. A minimum of 40 cells were analysed per condition in each experiment, *n* = 3 experiments. **P < 0.01, two-tailed *t* test. **B** RPE1 cells were transfected with siRNA control, siProtrudin #1 or siProtrudin #2. 96 h after transfection they were incubated with PS-biotin beads and processed as in (**A**). Micrographs show maximum intensity projections of z-stacks with arrows pointing to internalised beads. Protrudin-depleted cells have a lower number of internalised beads compared to the control. Graph shows the relative percentage of internalised beads to the total amount of cell-associated beads. Values denote mean ± s.d. A minimum of 50 cells was analysed per condition in each experiment, *n* = 3 experiments. ***P < 0.001, ****P < 0.0001, One-way ANOVA. siCtrl = siRNA control, siProt#1 = siRNA Protrudin oligonucleotide 1, siProt#2 = siRNA Protrudin oligonucleotide 2

To distinguish incomplete from complete bead uptake, we coated the PS-beads with a biotinylated lipid that could be detected by Alexa-488-conjugated Streptavidin (Streptavidin-Alexa-488) as long as the beads were not yet internalised. The internalised unlabelled beads were visible only in the brightfield channel. Upon 30 min of incubation, RPE1 OE Protrudin cells internalised a significantly higher portion of the beads than RPE1 cells (Fig. 7A). Conversely, when Protrudin was depleted using two independent siRNA oligos, the relative uptake of beads was strongly reduced (Fig. 7B, Supplementary Fig. 5B). These results support our previous findings and further indicate that the Protrudin pathway is important not only for the formation of the phagocytic cup, but also for the internalisation of the bound particle.

## Discussion

**Fig. 8:**
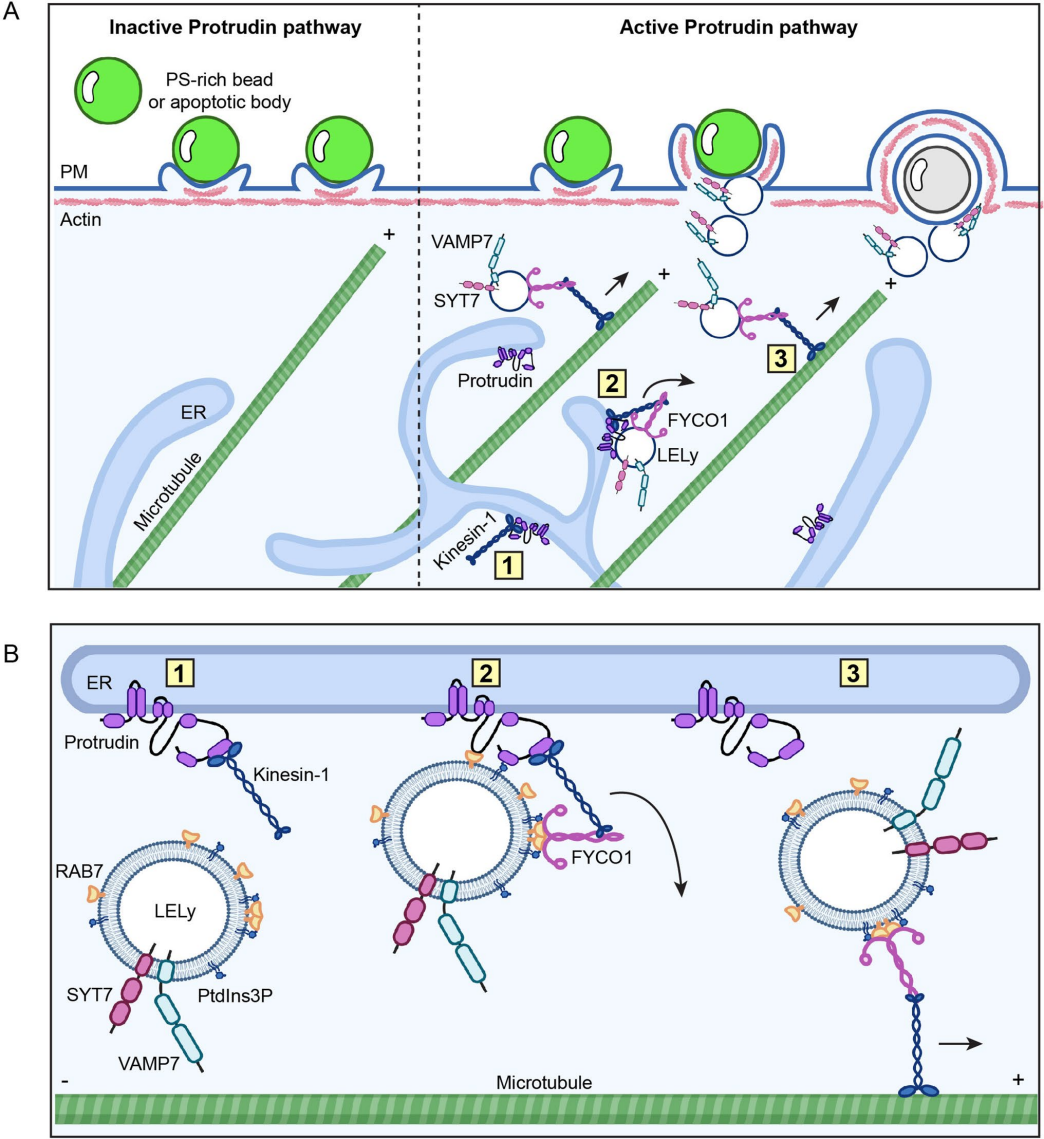
Model illustrating the role of the Protrudin pathway in phagocytosis. **A** Left: inactive Protrudin pathway leads to incomplete phagocytic cup formation. Non-successful uptake of PS-rich silica beads or apoptotic bodies (green sphere). Right: active Protrudin pathway results in efficient phagocytic cup formation and particle internalisation. The Protrudin pathway promotes Kinesin-1 dependent delivery of vesicles to the forming phagocytic cup. FYCO1 acts as an adapter protein between the motor protein Kinesin-1 and the RAB7 and PtdIns3P-positive vesicle. The endosomes fuse in a Ca^2+^-dependent manner with the PM mediated by SYT7 and VAMP7 to aid internalisation of particles. Internalised beads cannot be stained by Streptavidin-Alexa-488 and are visible as colourless spheres by brightfield microscopy (grey sphere, Fig. 7). Overexpression of Protrudin increases the cell’s capacity to internalise PS-rich particles. Created with BioRender.com. **B** Detailed illustration of Kinesin-1 handover from Protrudin to LELys at ER-endosome contact sites. (1) Kinesin-1 binds to Protrudin in the ER [67]. (2) Protrudin binds to RAB7 and PdIns3P at the LELy, forming contact sites. The endosomal adaptor protein FYCO1 receives Kinesin-1 [24]. (3) Kinesin-1 detaches from Protrudin and binds to a microtubule, resulting in plus end directed transport of the SYT7 and VAMP7-positive LELy. Created with BioRender.com

When the phagocytic cargo has a size of a few µm, stretching of the PM is not sufficient to sustain efficient internalisation [21, 32]. Endosomes contribute to membrane extension by fusing with the PM at the base of the phagocytic cup [15, 33], a process that requires microtubules [34, 35]. Here, we identify the Protrudin pathway as a major driver of microtubule-dependent endosome transportation in phagocytosis, stimulating phagocytic cup formation and phagocytic uptake in RPE1 cells (Fig. 8).

The Protrudin pathway mediates ER-endosome contact sites, where the anterograde microtubule motor protein Kinesin-1 gets loaded onto LELys by the help of the endosomal adaptor protein FYCO1 [24]. This facilitates transport of the endosomes along microtubules to the cell periphery where they fuse with the PM in a SYT7-dependent manner [24]. SYT7 is a Ca^2+^ binding protein which facilitates LELys exocytosis in cooperation with the endosomal SNARE-protein VAMP7 [20, 36]. Both proteins are required for phagocytosis [17, 19]. The late endosomal Ca^2+^ channel TRPML1 provides a local pool of Ca^2+^ and promotes focal exocytosis in phagocytic cup formation [18]. Our findings that FYCO1 localises to a population of VAMP7-positive LELys (Fig. 4B) and that the localisation of FYCO1 and VAMP7-positive vesicles in the cell periphery depends on Protrudin (Fig. 4A, B), strengthen the notion that Protrudin acts upstream of Ca^2+^-dependent exocytosis. Moreover, FYCO1 and VAMP7-co-positive vesicles were transported to the forming phagocytic cup during PS-bead internalization (Fig. 6A, B), and SYT7 depletion prevented the formation of phagocytic cups in Protrudin overexpressing cells (Fig. 3D). Taken together, this establishes the Protrudin pathway in the delivery of VAMP7/SYT7-positive LELys in phagocytosis and broadens our understanding of this process.

Several types of endosomes have been implicated in phagocytic cup formation. In addition to VAMP7-mediated exocytosis of LELys [17], VAMP3 and RAB11-positive recycling endosomes are involved [16, 37]. The Protrudin pathway engages FYCO1, which is recruited to LELys by binding to RAB7 and PtdIns3P [24]. We observed an enrichment of FYCO1 and RAB7-co-positive vesicles at the base of the cup (Fig. 2E, Supplementary Fig. 3B), in line with a role of the Protrudin pathway for the delivery of RAB7-endosomes. Although EEA1, which marks early endosomes, could be observed in close apposition to the cups (Fig. 2D, Supplementary Fig. 3B), this population of early endosomes was not enriched. Another cellular pathway for microtubule-dependent anterograde endosome translocation depends on BORC/SKIP and the late endosomal GTPase ARL8B [38, 39]. While ARL8B has been implicated in phagosome to lysosome trafficking and fusion [40, 41], it is not yet known whether an ARL8B-containing subpopulation of late endosomes also contributes to phagocytic cup formation.

The fact that not all cup-associated VAMP7-positive endosomes harboured FYCO1 could support the involvement of a RAB7-negative LELy subpopulation. However, it is conceivable that once the endosomes reach the PM, they undergo a phosphoinositide and RAB switch to prime them for PM-fusion [42–44]. With the loss of PtdIns3P and RAB7, FYCO1 will dissociate, whereas the transmembrane VAMP7 will remain. This is supported by our live cell imaging data, where VAMP7 and FYCO1-co-positive vesicles translocate to the base of the forming phagocytic cup, but only VAMP7 is detected in the growing cup, showing a diffuse PM localization as expected upon vesicle fusion with the PM (Video 1, Fig. 6B).

The contribution of internal membranes for phagocytic cup formation might differ in various cell types and likely depends on the size of particle being phagocytosed [19, 21, 45]. Most studies regarding endosomal membrane delivery in phagocytosis have been carried out in professional phagocytes, such as macrophages [17, 19, 35, 37, 45–48]. In Neutrophils, secretory granules can contribute to phagocytic cup formation and phagocytosis [34, 49]. Our finding that LELys provided by the Protrudin pathway are required for phagocytic cup formation and efferocytosis in RPE1 cells supports the existing results from professional macrophages. Moreover, our results suggest that the phagocytic cup is formed in a similar way in professional and non-professional phagocytes.

In RPE1 OE Protrudin cells, we observed an increased number of phagocytic cups, often more than one cup per cell, and more cells in the population had cups (Fig. 1B, Fig. 3C, D). This could be due to a stimulatory role in cup formation or an inhibition of cup resolution. Since these cells internalised more PS-beads (Fig. 7A), our results indicate that RPE1 OE Protrudin can stimulate cup formation, rather than stalling the process. This suggests that the contribution of internal vesicles from LELys might be a limiting factor in efferocytosis, supporting previous work on phagocytic capacity in macrophages [45, 48]. Studies using increasing phagocytic burden have indeed shown a corresponding increased dependency of microtubules, Kinesin-1 and SYT7 [19, 35, 45]. It has been suggested that the uptake of large particles requires membrane supply from large late endosomes, whereas the smaller recycling endosomes are sufficient to sustain the uptake of smaller particles [21]. We observed that the actin-rich cups have a diameter of 2–5 µm (Fig. 1C) consistent with the size of apoptotic bodies [27]. Apoptotic bodies might constitute a significant phagocytic burden [50] requiring supply from internal membranes, such as LELys, which can be delivered more efficiently in RPE1 OE Protrudin cells.

We cannot rule out that RPE1 OE Protrudin could contribute to stimulation of cup formation in additional ways. To probe the environment, phagocytic cells form filopodia-like protrusions, increasing the likelihood to capture potential cargo [51]. However, these cell extensions rely mostly on membrane stretching and actin, making it unlikely that the Protrudin pathway is involved in this very early phase of phagocytosis. Alternatively, mTORC1 activity has been implicated in phagocytosis [52, 53], and overexpressed Protrudin promotes mTORC1 signalling from peripherally localised LELys [54]. It is also tempting to speculate that Protrudin might increase the vesicle-mediated surface localisation of phagocytic receptors, such as TAM receptor tyrosine kinases and integrins [23, 55]. Moreover, Protrudin has a role in the shaping of the tubular ER network, which extends to the periphery of the cells [56]. ER-PM contact sites likely contribute to phagocytosis by increasing Ca^2+^ supplies upon high phagocytic burden [57, 58], and Protrudin could thus support a continued SYT7/Ca^2+^ dependent fusion of LELys with the PM. Indeed, Protrudin depletion strongly inhibited uptake of PS-coated silica beads (Fig. 7B). Taken together, our results are in line with a stimulatory role of the Protrudin pathway in phagocytosis.

This work establishes retinal pigment epithelial cells as an adequate cell culture system to study efferocytosis. By degrading shed photoreceptor discs, RPE cells maintain homeostasis in the eye, thereby preventing blindness [11, 12, 59, 60]. Our data support a role for the Protrudin pathway in this process. Interestingly, loss of FYCO1 is associated with autosomal-recessive cataracts [61, 62], and it is tempting to speculate that the function of the Protrudin pathway in efferocytosis could counteract the development of this eye disease.

## Materials and methods

### Antibodies

The following antibodies were used in this study: Mouse anti-β-Actin (Western blotting [WB] 1:5000, Sigma-Aldrich; A5316), mouse anti-FYCO1 (immunofluorescence [IF] 1:300, Abnova; H00079443-A01), rabbit anti-FYCO1 (IF 1:200, Invitrogen; PA5-45,805), mouse anti-GAPDH (WB 1:3000, Abcam; ab9484), rabbit anti-LAMP1 (IF 1:300, Merck Life Science; L1418), goat anti-mCherry (IF 1:200, OriGene; AB0040-200), rabbit anti-PARP (WB 1:1000, Bionordika; B9542S), rabbit anti-Protrudin (WB 1:7500, Protein Tech Group, 12680-1-AP), rabbit anti-RAB7 (IF 1:50–100, Cell Signalling Technology; D95F2), mouse anti-VAMP7 (IF 1:300, Synaptic Systems; 232 011), mouse anti-Vinculin (WB 1: 3000, Sigma; V9131), mouse-anti-Calnexin (IF 1:200, Abcam, ab22595), mouse anti-myc (IF 1:10, 9E10), and mouse-anti-GFP (IF 1:400, Merck Life Science 11814460001). The secondary antibodies were obtained from Jackson ImmunoResearch, Molecular Probes and LI-COR.

### Reagents

The following reagents were used: AnnexinV-Alexa-568 (IF 1:50, Invitrogen; A13202), Streptavidin-Alexa-488 (IF 2 µg/mL in PBS, Jackson ImmunoResearch Laboratories; 016-540-084), Phalloidin Rhodamine, Alexa Fluor 488 or Alexa Fluor 647 (IF 1:200, Molecular Probes; R415, A12379, A22287, respectively), Staurosporine (Sigma-Aldrich; S6942). The following lipids were used in this study: POPC, DOPS and DSPE-PEG (2000) Biotin all stored in Chloroform (Avanti Polar Lipids; 850457, 840035 and 880129, respectively).

### Plasmids

The pHluorin-Lamp1-mCherry construct is described in [24]. pDEST-mCherry-FYCO1 was a gift from Serhiy Pankiv and Terje Johannssen, Tromsø. pEGFP-VAMP7 was a gift from Thierry Galli (Addgene plasmid 42316).

### Cell culture and cell lines

The cell lines were grown according to ATCC instructions. The immortalised human retinal pigment epithelial (hTERT-RPE1) cell line (CRL-4000) was cultivated with DMEM/F12 with Glutamax (Gibco; 31331-093) supplemented with 10% fetal bovine serum (FBS) (Sigma; F7524), 100 U/mL penicillin and 0.1 mg/mL streptomycin from (Gibco; 15140130) at 37 °C with 5% CO_2_. Cell lines were authenticated by genotyping and regularly tested for mycoplasma contamination. The stable cell line RPE1 OE Protrudin (overexpressed Protrudin, resistant against Protrudin oligonucleotide #1) has been described in [25].

### siRNA transfections

All transfections were done using Lipofectamine RNAiMax (Invitrogen; 56532) according to the manufacturer’s protocol with 20 or 50 nm siRNA oligonucleotide per well. The following siRNA targeting sequences were used: siRNA Protrudin #1: 5′-AGAATGAGGTGCTGCGCAG-3′ (J-016349–12) [25], siRNA Protrudin #2: 5′-AACGGGTTCCTGAGCAAGAAT-3′ [25, 56], and siRNA SYT7: 5′-CCCTGAATGTCGAGGATAGTA-3′ [25, 63].

The siRNA oligonucleotides against Protrudin were from Horizon/Dharmacon (OnTargetPlus), and siRNA against SYT7 was from Ambion/Thermo Fisher Scientific (Silencer Select). As a negative control, non-targeting control siRNA was used (for Protrudin: Dharmacon; D-001810-01, for SYT7: AllStars (Qiagen); 1027281). Cells were analysed 48–96 h after transfection, as indicated in the figure legends, and the efficiency of the knockdown verified by WB for every individual experiment.

### Quantitative RT-PCR

Total RNA was extracted using RNeasy Plus mini kit (Qiagen; 74134). cDNA was synthesised using SuperScript IV Reverse Transcriptase (Thermo Fisher Scientific; 18090010). Quantitative PCR was performed using the cDNA, SYBR Green I Master Mix (Roche; 04707516001), LightCycler 480 (Roche), and QuantiTect Primer Assays (QT00086975 for SYT7 and QT00000721 for TATA-binding protein [TBP]; Qiagen). Cycling conditions were 5 min at 95 °C followed by 45 cycles for 10 s at 94 °C, 20 s at 56 °C, and 10 s at 72 °C. A standard curve made from serial dilutions of cDNA was used to calculate the relative amount of the different cDNAs in each sample. SYT7 expression was normalised to the expression of the internal standard TBP.

### Immunoblotting/Western blotting

The cells were washed three times in ice-cold PBS before being lysed in 2 × sample buffer (125 mM Tris–HCl, pH 6.8, 4% SDS, 20% glycerol, 0.004% bromophenol blue supplemented with 200 mM DTT). The proteins were separated by SDS-PAGE on 10% or 4–20% gradient TGX Precast gels (Bio-Rad; 567–1034 or 567–1094, respectively) and blotted on PVDF membranes (Bio-Rad; 170–4273, 170–4274). Membranes were visualised using the fluorescently labelled secondary antibodies (IRDye680 and IRDye800, LI-COR) and scanned by Odyssey Developer (LI-COR).

### Immunostaining

Cells were grown on coverslips and prepermeabilised in 0.05% saponin in PEM (0.1 M Pipes, (Sigma-Aldrich; P7643), 2 mM EGTA (Sigma; E3889), and 1 mM MgSO_4_ (Merck Millipore; 105886), pH 6.95) buffer for 5 min on ice before fixation to reduce the cytosolic pool of proteins [64], or directly fixed with 3% formaldehyde for 15 min at room temperature (on ice for phagocytic uptake experiments). Cells were then washed three times with PBS and once in 0.05% saponin diluted in PBS. Proteins were stained with primary antibodies for 1 h and washed three times in PBS/saponin before they were stained with secondary antibodies for 45 min. The coverslips were mounted with Mowiol containing 2 µg/mL Hoechst 33342 (Thermo Fisher Scientific; H3570) or ProLong Diamond Antifade Mountant with DAPI (Invitrogen; P36966). For the detection of PS on apoptotic bodies, cells were directly fixed and then, incubated with AnnexinV-Alexa-568 overnight at 4 °C, before they were stained with secondary antibodies and mounted as described. For experiments using Streptavidin-Alexa-488, coverslips were stained for 4 min on ice and washed before fixation to avoid the stain from leaking into the cell after fixation.

### Liposome generation and silica bead coating

Liposomes were generated with a modified protocol as previously published [65]. The lipid stock solutions of POPC and DOPS were mixed in a molar ratio of 90/10 mol % or by mixing POPC, DOPS and DSPE-PEG (2000) Biotin in a molar ratio of 89.5/10/0.5 mol %. The lipid mix was dried under N_2_ gas to a thin film before it was placed under vacuum for 45 min. The film was rehydrated in a HEPES-based buffer (Live Cell Imaging Solution, Invitrogen; A14291DJ0) in room temperature and vortexed for 1 min. The suspension was frozen and thawed five times using liquid N_2_ and a 37 °C water bath before it was extruded by 0.1–0.2 µm pore size filter 11 times. The liposomes were stored at 4 °C and used within 4 weeks.

To coat beads with liposomes, 50 µL of 4 µm silica beads in suspension (Bangs Laboratories; SS05002) were washed three times in 300 µL MilliQ. Silica beads were coated by adding 60 µL of liposome suspension and 240 µL of Live Cell Imaging Solution (Invitrogen; A14291DJ0) by rotation in room temperature for 45 min. Beads were then washed gently three times and used within 24 h.

### Generation of apoptotic bodies

Cells were seeded in 10 cm dishes (one dish yields apoptotic bodies for one well in a 24-well plate) in complete medium overnight. Medium was replaced with 1 μM Staurosporine in serum-free medium and incubated for 17–20 h. The medium was collected, and the dish was gently washed with PBS once to collect the apoptotic bodies. Medium and PBS was pooled and spun down at 300*g* × 10 min to remove dead cells. The supernatant was centrifuged at 3000*g* × 20 min to get the apoptotic body fraction. The pellet was resuspended in 1 mL complete culture medium. The apoptotic bodies were used within 24 h after harvest. An antibody against full-length and cleaved PARP was used to assess apoptosis.

### Phagocytosis assays

#### Liposome coated beads

Cells in culture dishes were precooled on ice before addition of the liposome-coated silica bead slurry. To allow phagocytic uptake for a defined period of time, the plate was incubated at 37 °C for 15 min for the analysis of vesicle recruitment and 30 min for the bead uptake assay. Cells were gently washed immediately after the indicated time to remove unbound beads and then, depending on the experiment, cells were either prepermeabilised, directly fixed or prestained with Streptavidin-Alexa-488 before fixation. PS-bead uptake was visualised by Zeiss LSM 880 confocal microscopy with 60× oil objective at random locations.

#### Apoptotic bodies

Cells were seeded on coverslips in a 24-well plate in complete medium. The next day medium was replaced with medium containing apoptotic bodies and incubated for 15 min at 37 °C. Cells were fixed and stained with Phalloidin Rhodamine to visualise the actin structures.

### Quantification of PS-bead uptake

Confocal z-stacks of minimum 40 bead-associated cells were captured per condition, per experiment from three experiments. Bead uptake was quantified by manually counting Streptavidin-Alexa-488-positive or -negative beads located within cell boarders (actin). Quantifications were presented as the percentage of internalised beads to the total amount of beads counted.

### Quantification of phagocytic cups

Phalloidin was used to visualise actin filaments in the phagocytic cups. The number of phagocytic cups was quantified from confocal z-stacks obtained at random locations on the coverslips. Actin rings were counted manually from the micrographs with criteria of being hollow, having a certain diameter and being in at least three confocal planes (1 μm high) on the dorsal side of the cell above actin stress fibres. To study the typical diameter of phagocytic cups, all cups wider than 1 μm were measured. For the quantification of cups per cell, the diameter had to be between 2.0 and 5.0 μm. Diameters were measured in Fiji using the straight-line tool, measuring the space between the outer boarders of the cup at its widest point.

### Confocal fluorescence microscopy and image analysis

Confocal micrographs were captured by Zeiss LSM 780, Zeiss LSM 880 Airyscan (Carl Zeiss) microscopes using a Zeiss plan-apochromat 63× NA/1.40 oil DIC II objective (Carl Zeiss) or Nikon Ti2-E with a plan-apochromat × 40 NA/0.95 air DIC N2 objective. Images were processed in ImageJ/Fiji [66] to adjust brightness and contrast and analysed as described. 3D surface rendering was done in Imaris version 9.0.2. (Bitplane). A minimum of five images, but often more, were taken of each condition from each experiment at random positions throughout the coverslips. All images within one dataset were taken at fixed intensities below saturation, with identical settings applied for all treatments within one experiment.

### Analysis of VAMP7 and FYCO1 vesicle positioning and colocalization

The NisElements software was used for fluorescence intensity-based segmentation of VAMP7- and FYCO1-positive dots from confocal micrographs. Hoechst-positive nuclei were used to segment nuclei. The perinuclear area was defined as an 8 µm broad circular area around the nucleus. The sum intensity of VAMP7 only dots or the number of VAMP7 and FYCO1 co-positive dots were automatically quantified in the perinuclear area, and in the whole cell, and the perinuclear positioning of dots was represented as % of the total population of dots. The Pearson correlation coefficient for VAMP7, RAB7, EEA1, LAMP1 and FYCO1 was calculated using the JaCoP plugin in ImageJ.

### Statistical analysis

The number of individual experiments and the number of cells or images analysed are indicated in the figure legends. For parametric data, an unpaired two-sided *t* test was used to test two samples with equal variance, and a one-sample *t* test was used in the cases where the value of the control sample was set to 1 or 100. For more than two samples, we used one-way ANOVA with Dunnet's post hoc test. All error bars denote mean values ± s.d., as indicated in every figure legend (*P < 0.05; **P < 0.01; ***P < 0.001). No samples were excluded from the analysis.

### Supplementary Information

Below is the link to the electronic supplementary material.

Supplementary file1 (PDF 5652 KB)

Supplementary file2 Video 1 Live-cell imaging of RPE1 cells transfected with GFP-VAMP7 and mCherry-FYCO1. PS-beads were added immediately before starting image acquisition. Co-positive endosomes are moving to the forming phagosome. Left panel: Close-up of a PS-bead as it is engulfed showing endosomes accumulating at the base of the phagocytic cup. Right panel: Coloured lines represent the transportation routes of double-positive endosomes to the phagocytic cup. (MP4 4434 KB)

Supplementary file3 Video 2 Live-cell imaging of RPE1 cells transiently transfected with pHluorin-LAMP1-mCherry. Immediately before filming PS-beads were added. Video displays a cell that engulfs three beads that gradually become acidified, indicating phagosome maturation. Newly formed phagosomes are pHluorin-positive due to neutral pH, while mature phagosomes have low pH and are mCherry-positive. (MP4 2185 KB)

## Data Availability

Primary data will be available from the authors upon request.
