# Supplementary material for: Protrudin-mediated ER-endosome contact sites promote phagocytosis
Source: Cell Mol Life Sci. 2023 Jul 19;80(8):216. doi: 10.1007/s00018-023-04862-0 (PMC10356898; doi:10.1007/s00018-023-04862-0)
Supplement: Supplementary file 1 — Supplementary file1 (PDF 5652 KB) [file 18_2023_4862_MOESM1_ESM.pdf]

Supplementary Information

Protrudin-mediated ER-endosome contact sites promote phagocytosis

Elfmark et al. 2023
Cellular and Molecular Life Sciences

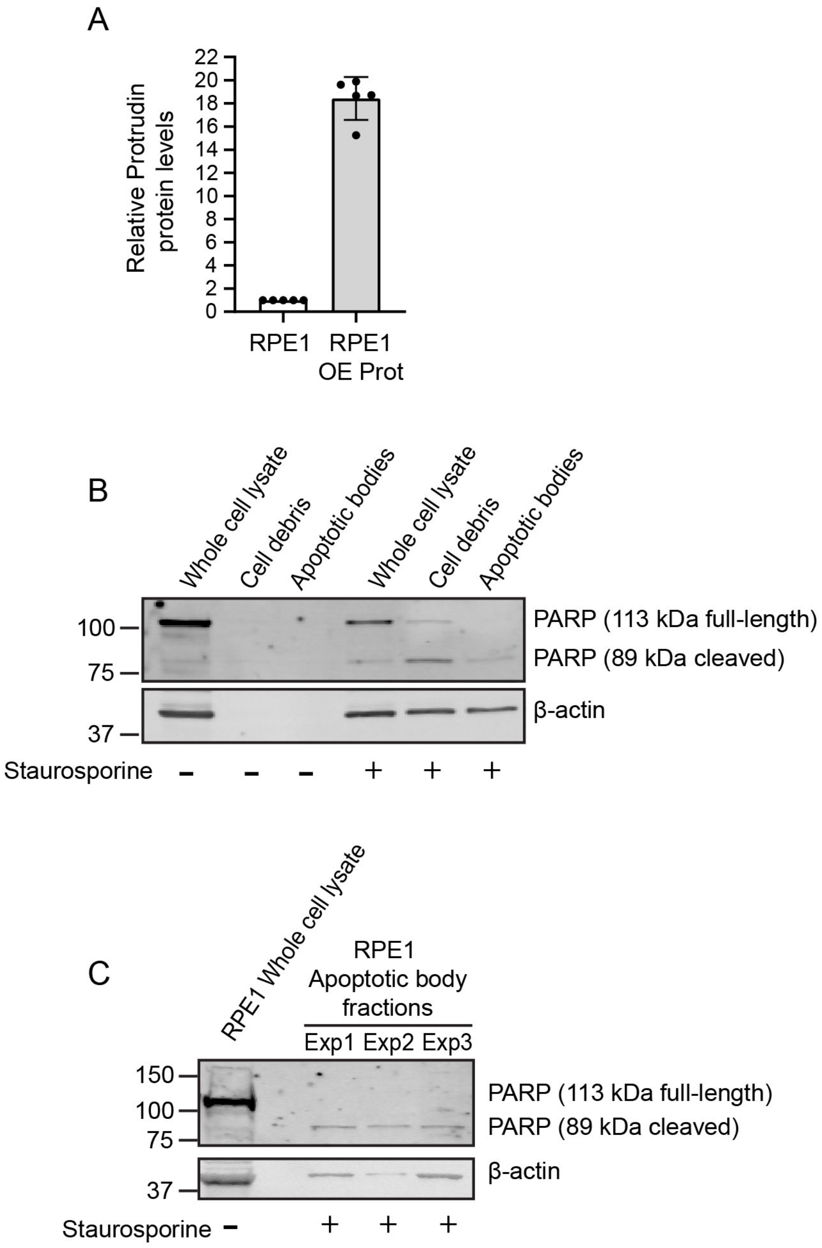

Supplementary Figure 1. Western blot validation of Protrudin expression and induced apoptosis in lysates from apoptotic bodies

A) Graph representing the quantification of the relative level of Protrudin expression in the indicated cell lines.

B) Western blot showing whole cell lysate, cell debris fractions (300*g* centrifugation) as well as apoptotic body fractions (3000*g* centrifugation) from RPE1 cells treated with DMSO or 1 µM Staurosporine for 24 hours. Note that the DMSO treated cells do not generate cell debris or apoptotic bodies, whereas Staurosporine treatment induces apoptosis as detected by cleavage of PARP in all fractions. β-actin was used as loading control.

C) RPE1 cells were treated with 1 µM Staurosporine for 24 hours to induce apoptosis before lysates were collected from each biological experiment, n = 3 experiments (corresponding to dataset in Fig. 1F). Cleaved PARP (89 kDa), but not full-length PARP (113 kDa) was detected with Western blotting in the apoptotic fractions, demonstrating successful induction of apoptosis. β-actin was used as loading control.

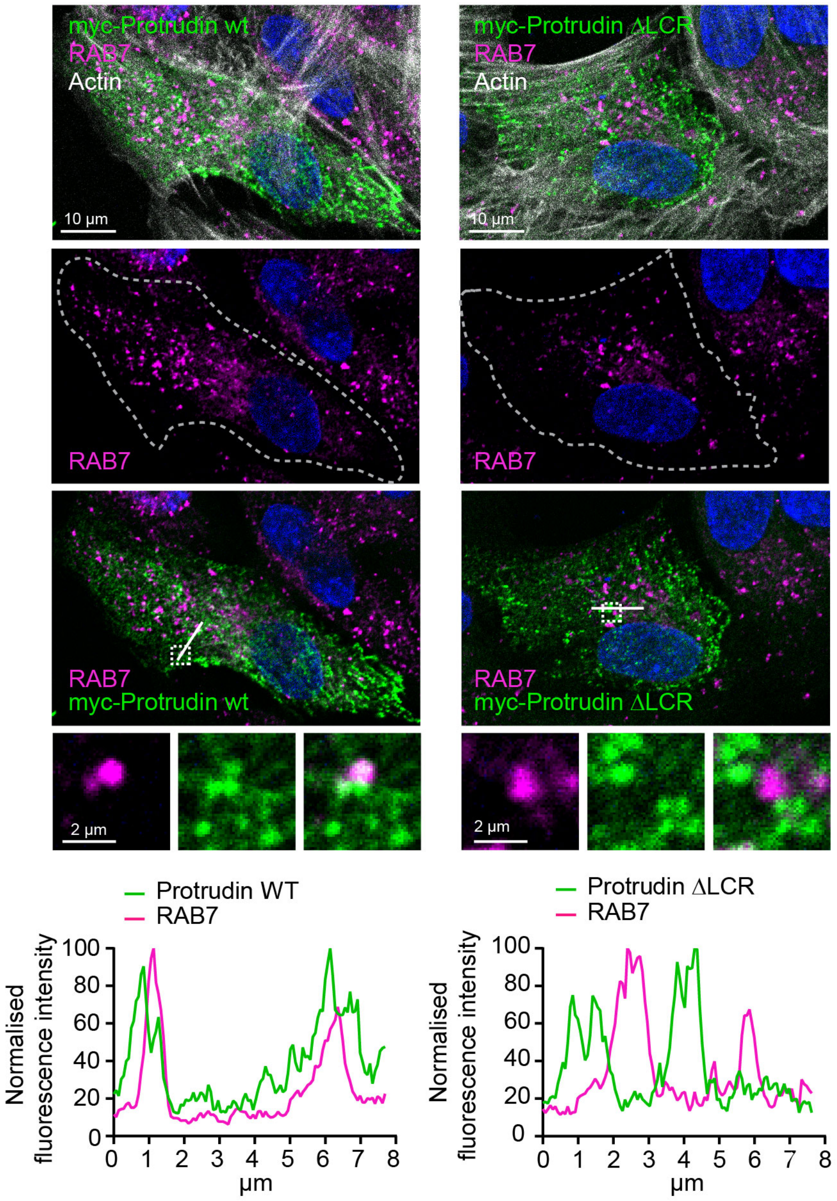

Supplementary Figure 2. RAB7 binding-deficient Protrudin fails to form ER-endosome contact sites

RPE1 cells were transiently transfected with myc-Protrudin wt or a RAB7 binding-deficient deletion mutant, myc-Protrudin ∆LCR. The insets and fluorescence intensity line plots show that RAB7-positive endosomes are making contact with Protrudin wt but not with Protrudin ∆LCR. As a result, the RAB7 endosomes have a more perinuclear localization in Protrudin ∆LCR expressing cells.

Elfmark et al., Supplementary Figure 3

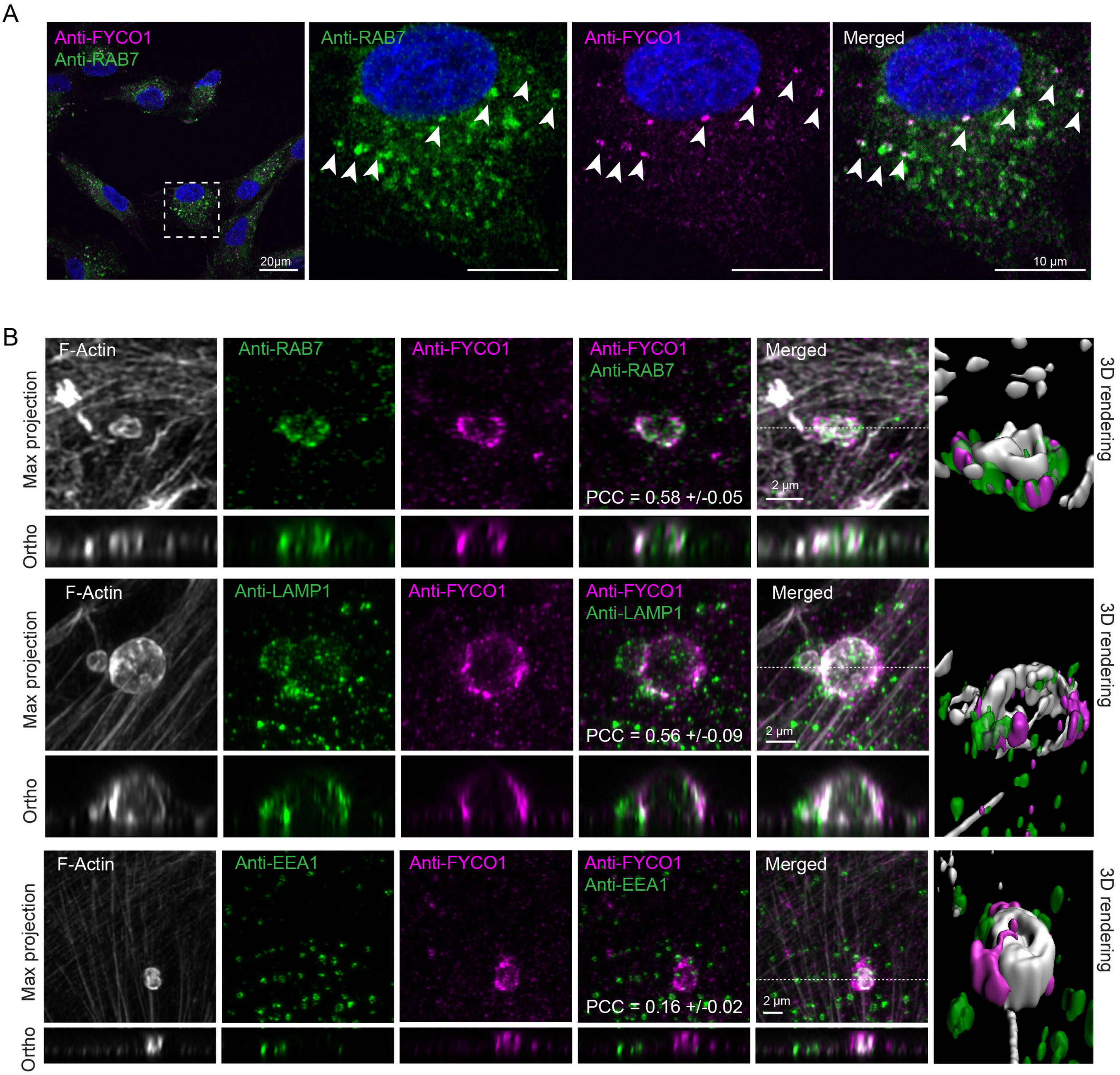

Supplementary Figure 3. FYCO1, RAB7 and LAMP1 endosomes are enriched in phagocytic cups

A) RPE1 cells were grown on coverslips, immunostained with anti-RAB7 and anti-FYCO1 antibodies and analysed with confocal microscopy. Consistent with previous reports using other cell lines [24, 25], FYCO1 localises to RAB7-positive endosomes. Arrowheads in the insets indicate colocalisation. Shown are representative images from 5 micrographs taken per experiment, n = 2.

B) RPE1 cells were immunostained with anti-FYCO1 in combination with either anti-RAB7, anti-LAMP1 or anti-EEA1 antibodies. F-actin is labelled with Phalloidin. Maximum intensity projections of confocal z-stacks obtained with Airyscanning show the actin cup and the indicated endosome-markers. Bottom panels display the structure from an orthogonal view. The FYCO1 signal is enriched together with RAB7 and LAMP1-positive endosomes, but not EEA1-positive endosomes. Pearson correlation coefficient (PCC) +/- s.d. from n = 4 or 5 images are shown for the different marker combinations. A 3D rendering shows the structures from a tilted side view.

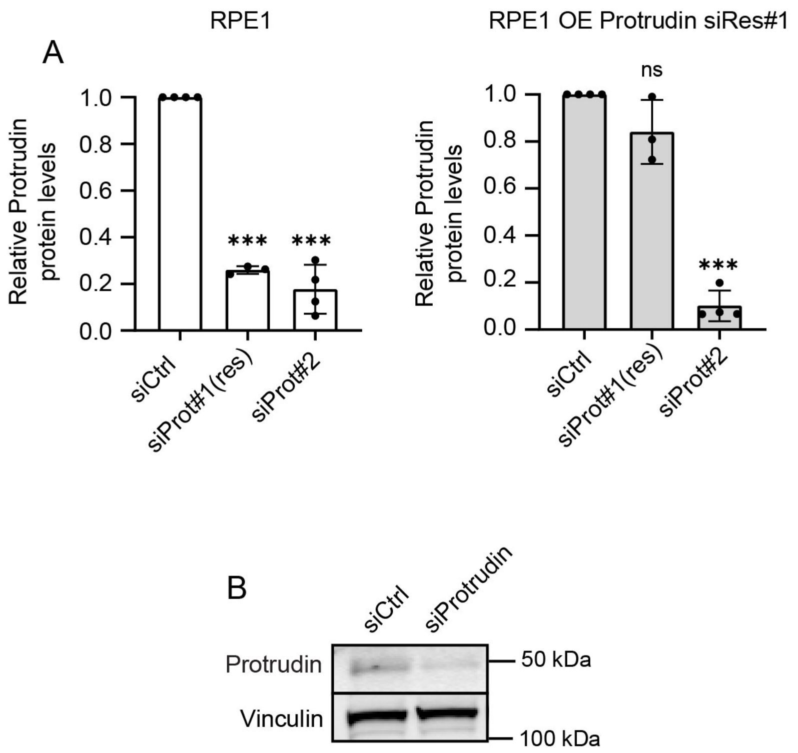

Supplementary Figure 4. Verification of Protrudin knock down

A) Quantification of the relative level of Protrudin expression from 3-4 independent experiments. Representative WB example in Fig. 3A. Error bars denote mean +/- s.d., ***P<0.001, ns = not statistically significant, One sample *t*-test.

B) Western blot demonstrates Protrudin knockdown efficiency from lysates of one representative experiment for Fig. 4.

Elfmark et al., Supplementary Figure 5

A

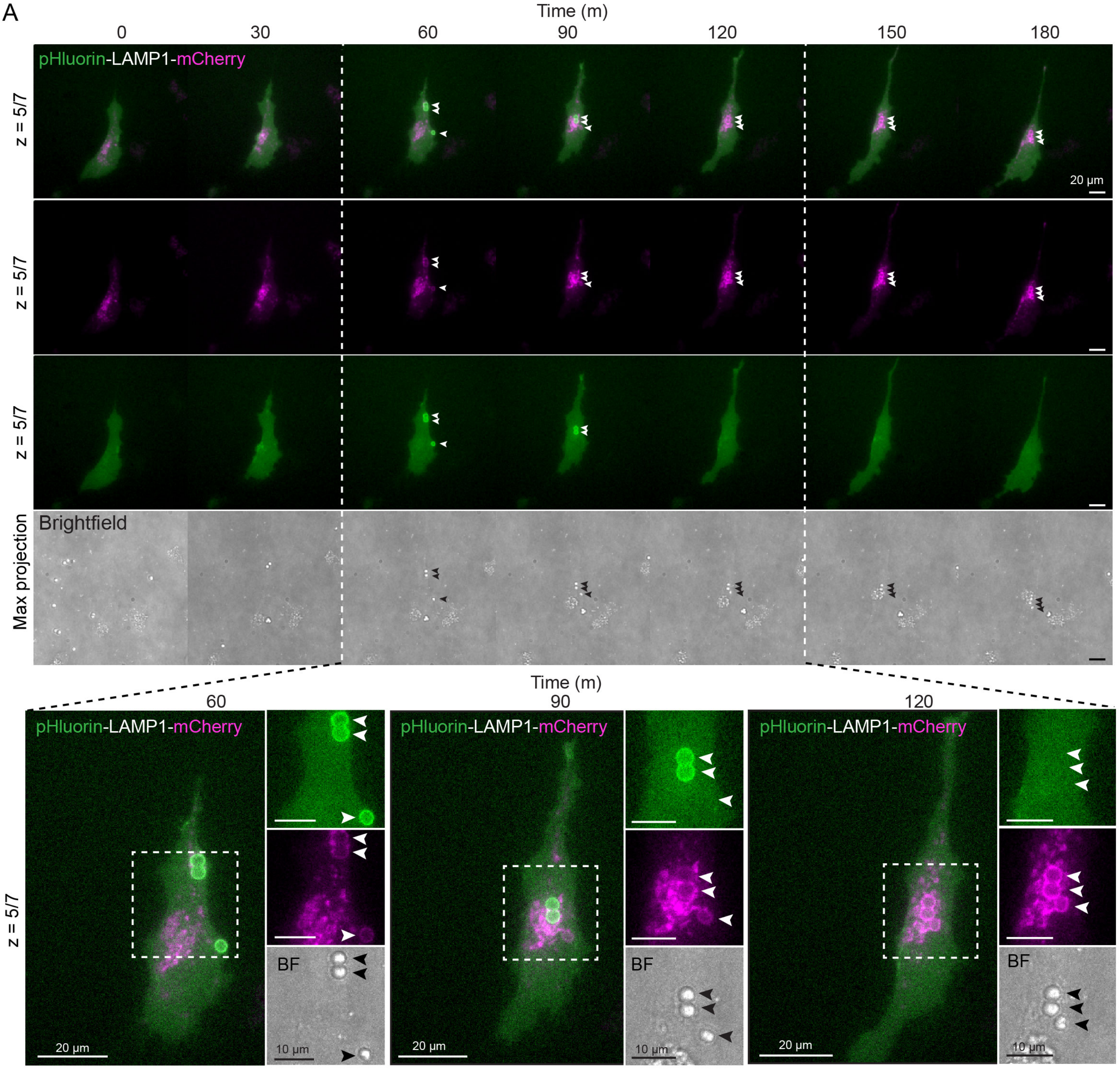

B

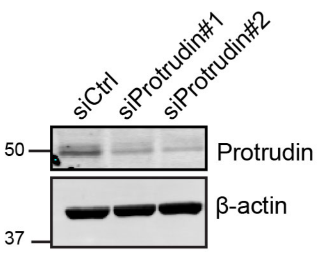

Supplementary Figure 5. PS-beads become internalised, trafficked and acidified in RPE1 cells

A) RPE1 cells were transfected for 24 hours with pHluorin-LAMP1-mCherry before live imaging in the presence of PS-beads (still images from Video 2). The construct has a pH-sensitive version of GFP (pHluorin) and a pH-stable mCherry tag to visualise both neutral (green/white) and acidic (magenta) compartments by quenching pHluorin at low pH. Montage shows a cell that internalises three PS-beads that gradually become more acidic. Bottom images focus on the beads between minute 60 and 120. “z =” indicates which optical confocal section from the z-stack is displayed.

B) RPE1 cells were transfected with control siRNA, or siRNA oligonucleotides against Protrudin for five days. Lysates of cells were harvested to validate Protrudin knockdown efficiency by Western blotting, using β-actin as loading control. Representative Western blot from one of three experiments (corresponding to Fig. 7B), n = 3. siCtrl = siRNA control, siProtrudin#1 = siRNA Protrudin oligonucleotide 1, siProtrudin#2 = siRNA Protrudin oligonucleotide 2.
